# Use of Impella heart pump for management of women with peripartum cardiogenic shock

**DOI:** 10.1002/clc.23249

**Published:** 2019-08-22

**Authors:** Uri Elkayam, Andreas Schäfer, Alaide Chieffo, Alexandra Lansky, Shelley Hall, Zoltan Arany, Cindy Grines

**Affiliations:** ^1^ University of Southern California Los Angeles California; ^2^ Hannover Medical School Hannover Germany; ^3^ San Raffaele Scientific Institute Milan Italy; ^4^ Yale School of Medicine New Haven Connecticut; ^5^ Baylor University Medical Center Dallas Texas; ^6^ University of Pennsylvania Philadelphia Pennsylvania; ^7^ Northwell Health Manhasset New York

**Keywords:** heart failure, Impella heart pumps, mechanical circulatory support, peripartum cardiomyopathy

## Abstract

**Background:**

Percutaneous mechanical circulatory support (MCS), such as the Impella heart pump is a valuable option for cardiogenic shock (CS), although the use of Impella in CS due to peripartum cardiomyopathy (PPCM) is limited.

**Objective:**

To assess outcomes in women with PPCM supported with an Impella device from the global catheter‐based ventricular assist device (cVAD) Registry.

**Methods and Results:**

A total of 15 women with PPCM supported with Impella devices between November 2008 and October 2015 were included. Of the 15 women, five were treated at Hannover medical school and have been reported previously, the rest were managed at various US hospitals. The mean age was 30.0 ± 7.34 years, eight women were Caucasian, and seven were African‐American. The occurrence of PPCM was post‐delivery in eight (53.3%), at delivery in one (6.7%), and during gestation in four women (26.7%). At admission, all women had severe heart failure with a mean ejection fraction of 14.7 ± 6% and 13 women (86.7%) presented with CS. Prior to Impella, 100% were mechanically ventilated, 79% received inotropes/vasopressors, 20% supported with IABP, and 27% received veno‐arterial extracorporeal membrane oxygenation (VA ECMO) during Impella support. Two women (13.3%) died, and 13 (87.7%) survived to discharge. Eight women (53.3%) had a recovery of native heart function and six (40%) were bridged to durable left ventricular assist device (LVAD).

**Conclusion:**

MCS with Impella devices can be successfully used as a bridge to early improvement, heart recovery, or successful implantation of durable LVAD in women with PPCM complicated by severe LV dysfunction.

## 1 INTRODUCTION

Peripartum cardiomyopathy (PPCM) is a myocardial disease characterized by left ventricular (LV) systolic dysfunction (LV ejection fraction < 45%) and heart failure, presenting typically in the later part of pregnancy or the first few months postpartum.^1^ The incidence of PPCM varies from 1:100 to 1:3000 depending on race and geographic regions and appears to be increasing, possibly due to rising maternal age, increased rate of multifetal pregnancies, and increased recognition of the disease.^2^ PPCM can be associated with severe complications including cardiogenic shock (CS), cardiac arrest, and death.^2^, ^3^ A recent meta‐analysis of 46 studies from 13 countries on women with PPCM reported a global mortality rate of 9%.^4^

Although recovery of LV function is frequently observed, the continued deterioration of cardiac function is reported in about 5%‐20% and can lead to severe heart failure, cardiogenic shock, and mortality.^3^ Early identification and hemodynamic stabilization is needed to increase the likelihood of myocardial recovery, particularly in PPCM complicated by CS. While medical management using catecholamines may be considered, their effect may be limited in patients with CS, and their use is often associated with adverse events effects including tachycardia, hypotension, myocardial ischemia, and arrhythmias.^5^

Percutaneous mechanical circulatory support (MCS) devices are a promising therapeutic option given their ability to improve hemodynamics and tissue perfusion.^6^ Recent reports suggest significant recovery with MCS in patients with CS due to PPCM not responding to medical therapy. The current American and European guidelines suggest early use of MCS in patients with CS.^7^, ^8^ The Impella devices (Abiomed, Danvers, Massachusetts) are transvalvular microaxial pumps that can be rapidly deployed in the catheterization lab and provide LV unloading with forward blood flow of up to 5 L/min.^6^ Given the low prevalence, published reports on the use of Impella devices in PPCM is limited.^9^, ^10^ We report on 15 women with PPCM supported with an Impella device from the global catheter‐based ventricular assist device (cVAD) Registry. This study, to our knowledge, is the largest reported series to date on the use of Impella devices in women with severe heart failure and CS due to PPCM.

## 2 METHODS

### 2.1 Study population

The current study is a retrospective analysis of 15 patients with PPCM undergoing LV unloading with Impella devices between November 2008 and October 2015, identified in the global cVAD registry. Of the 15 patients, five patients were treated at Hannover medical school (a cVAD site) and have been previously described.^10^ The remaining patients were enrolled at eight cVAD sites within the United States. The global cVAD registry is an ongoing registry of patients who received Impella support or attempted support in North America and Europe since the inception of the registry in 2009 to date.^11^ The cVAD Registry was designed by an Executive Steering Committee that oversees its ongoing conduct. The registry protocol was reviewed and approved by the Institutional Review Board at each participating site. Sites are invited to report all consecutive Impella cases without pre‐selection of indication or patients. In order to avoid patient selection bias, a process was developed to reconcile the site's utilization of the device (commercial database) with reporting in the clinical cVAD registry. Patients that were identified as having received an Impella device in the commercial database were expected to be reported in the cVAD Registry database; otherwise, sites were notified of the obligation to enter and report the cases to ensure consecutiveness.

### 2.2 Patient treatment and follow‐up

Data were abstracted from the medical record to a standard electronic case report form by the sites study coordinators who were centrally trained. Information was collected on the patient's demographic characteristics, medical history, clinical presentation, hemodynamic, echocardiographic, and angiographic characteristics, treatment during hospitalization, hospital discharge status, and follow‐up status.

CS was defined as hypotension (systolic blood pressure < 90 mm Hg or need for inotropes/vasopressors to maintain systolic blood pressure > 90 mm Hg) and/or end‐organ hypoperfusion indicated by altered mental status, clammy skin, or lactate >2 mmol/L after adequate correction of preload.^10^ All patients were admitted to the intensive care unit where hemodynamic support with an Impella device was initiated. Impella devices were implanted via either femoral or axillary access and the correct position was verified every 8 hours by transthoracic echocardiography. Hemodynamic measurements were performed using a Swan‐Ganz catheter. Clinical decisions regarding device repositioning due to reduced blood flow, device explanation, and early weaning of device were made by the treating physician.

In cases of additional right heart failure (central venous pressure > 18 mm Hg and cardiac index <1.5 L/min/m^2^), a venous arterial extracorporeal membrane oxygenation (VA‐ECMO) was implanted via femoral venous and arterial access. In all patients receiving VA‐ECMO, antegrade leg perfusion was enabled with a 6F sheath to prevent ischemia. All patients on MCS received unfractionated heparin for anticoagulation with an activated clotting time of 160 to 180 seconds.

Patients were treated according to current guidelines^5^ and institutional standard of care for acute heart failure and CS. Five patients (from Hannover cohort) also received the prolactin inhibitor bromocriptine, according to the bromocriptine treatment scheme for women with PPCM and CS from Hannover Medical school.^10^ Bromocriptine dosage was adjusted based on sequential measurements of prolactin levels (up to 10 mg twice a day, if necessary). In these five patients, levosimendan was also administered on day 1 and days 7 to 10. After hemodynamic stabilization and removal of percutaneous MCS, medical therapy for chronic heart failure was initiated, including stepwise beta‐blockers, angiotensin‐converting enzyme (ACE) inhibitors, mineralocorticoid antagonists (MRAs), and ivabradine. Patients with reduced left ventricular ejection fraction (LVEF) at discharge were provided with a wearable cardioverter/defibrillator (WCD) to prevent sudden cardiac death. Patients were followed up in PPCM outpatient clinics as per institutional routine care, usually at 1, 6, and 12 months after discharge.

The diagnosis of PPCM was based on the criteria established by the European Society of Cardiology Working Group in 2010 that defined PPCM as idiopathic cardiomyopathy presenting with HF secondary to LV systolic dysfunction towards the end of pregnancy or in the months following delivery, where no other cause of HF is found.^11^

Recovery was defined as successful weaning of Impella, followed by survival to discharge without the need for additional MCS support or heart transplant.

### 2.3 Statistical analysis

Data are represented as n (%), mean ± SD for variables with normal distribution or median and range (minimum‐maximum) for non‐normal distribution. Change in hemodynamics before and during Impella support and in LVEF from baseline to discharge and post‐discharge follow‐up was analyzed using the paired *t* test. A two‐sided *P*‐value of less than .05 was considered statistically significant and all statistical tests were performed using SAS version 9 (SAS Institute, Cary, North Carolina) software.

## 3 RESULTS

### 3.1 Patient characteristics

Between November 2008 and October 2015, 15 women with PPCM treated with an Impella device were enrolled. Of the 15 women with PPCM, eight were Caucasian and seven were African‐American (Table 1). The occurrence of PPCM onset was during gestation in 26.7% (4/15) (range 23‐38 weeks of gestation), at delivery in 6.7% (1/15), post‐delivery in 53.3% (8/15) (range 5 days to 6 months), and unknown in 13.3% (2/15) (Table 2). About 69% of the patients (9/13) were transferred from another hospital (Table 3) and 100% (7/7) were mechanically ventilated. The mean age was 30.0 ± 7.3 years and 40% were primigravida (4/10).

**Table 1:** Patient and baseline characteristics

Characteristics		N
Age, year	30.0 ± 7.3	15
Race		
Black or African‐American	7 (46.7%)	15
Caucasian	8 (53.3%)	15
Height, cm	165.0 ± 7.5	9
Weight, kg	77.7 ± 17.2	9
BMI, kg/m^2^	27.4 ± 6.1	13
Smoker	3 (20%)	15
Hypertension	3 (20%)	15
Diabetes mellitus	1 (6.7%)	15
Coronary artery disease	0 (0%)	14
Liver insufficiency	2 (22.2%)	9
COPD/chronic pulmonary disease	0 (0%)	15
Arrhythmia	1 (10%)	10
Prior AICD/pacer implanted	1 (10%)	10
**Hemodynamics prior to Impella support**		
Heart rate, beats per minute	114 ± 25.7	15
Mean arterial pressure, mm Hg	75 ± 18.7	15
Cardiac index, L/min/m^2^	2.0 ± 0.3	4
Cardiac output, L/min	3.7 ± 0.8	4
PCWP, mm Hg	29.7 ± 10.2	7
Left ventricular ejection fraction, %	14.7 ± 6.1	15
**Hematology and blood chemistry pre‐support**		
Red blood cells, 10^6^/μL	3.9 ± 0.7	9
White blood cells, 10^3^/μL	13.1 ± 8.1	10
Hemoglobin, g/dL	10.7 ± 2.1	10
Hematocrit, %	32.7 ± 5.9	10
Platelet count, 10^3^/μL	243.2 ± 136.5	10
BNP, pg/mL	1347.2 ± 540.2	5
NT‐proBNP, pg/mL	844	1
ACT, seconds	186.5 ± 103.9	2
INR	1.7 ± 1.5	9
Total bilirubin, mg/dL	0.84 ± 0.7	8
Creatinine, mg/dL	1.0 ± 0.7	10
BUN, mg/dL	18.4 ± 15.1	5
GFR, mL/min/m^2^	57.5 ± 13.1	6

*Note*: Data presented as mean ± SD, or n (%).

Abbreviations: ACT, activated clotting time; AICD, automatic implantable cardioverter‐defibrillator; BMI, body mass index; BNP, brain natriuretic peptide; BUN, blood urea nitrogen; COPD, chronic obstructive pulmonary disease; GFR, glomerular filtration rate; INR, international normalized ratio; NT‐proBNP, N‐terminal pro‐B‐type natriuretic peptide; NYHA, New York Heart Association, PCWP, pulmonary capillary wedge pressure.

**Table 2:** Peripartum cardiomyopathy characteristics

Characteristics		N
Timing of PPCM onset		
During gestation	4 (26.7%)	15
At delivery	1 (6.7%)	15
Post‐delivery	8 (53.3%)	15
Unknown or not documented	2 (13%)	15
Total number of gestations		
1	4 (40%)	10
2	1 (10%)	10
3	1 (10%)	10
4	2 (20%)	10
>4	2 (20%)	10
Eclampsia or pre‐eclampsia	1 (16.7%)	6
Delivery		
Vaginal	4 (30.7%)	13
Caesarean section	9 (69.2%)	13
Labor		
Natural	2 (40%)	5
Induced	3 (60%)	5
Alive newborn	6 (85.7%)	7
Timing of Impella pump insertion		
Prior to labor	1 (9.09%)	11
During labor	0 (0%)	11
After labor	10 (90.9%)	11

*Note*: Data presented as n (%).

**Table 3:** Admission, procedural, and on‐support characteristics and outcomes

Characteristics		N
**Prior to Impella support**		
Patient transferred from another hospital	9 (69.2%)	13
NYHA Class III/IV	8 (100%)	8
Cardiomyopathy	15 (100%)	15
Shock prior to device implant	13 (86.7%)	15
Duration of shock	50.3 ± 75.5	10
<6 hours	3 (23.1%)	13
6‐12 hours	3 (23.1%)	13
12‐24 hours	1 (7.7%)	13
>24 hours	5 (38.5%)	13
Unknown or not documented	1 (7.7%)	13
If shock, patient experienced any of the following		
Anoxic brain damage	2 (33.3%)	6
End‐organ hypoperfusion	2 (28.6%)	7
Cardiac arrest	2 (16.7%)	12
Out‐of‐hospital cardiac arrest	1 (10%)	10
Witnessed	1 (10%)	10
Return of spontaneous circulation	1 (10%)	10
In‐hospital cardiac arrest prior to Impella	0 (0%)	10
Return of spontaneous circulation	0 (0%)	10
If shock, patient required any of the following		
Mechanical ventilation	7 (100%)	7
Cardiopulmonary resuscitation	2 (28.6%)	7
Evidence of right ventricular failure	3 (42.9%)	7
Inotropes/vasopressors	11 (78.6%)	14
Maximum number of different inotropes	2.55 ± 1.04	11
IABP support	3 (20%)	15
**During Impella support**		
Impella device type		
Impella 2.5	5 (33.3%)	15
Impella CP	8 (53.3%)	15
Impella 5.0	2 (13.3%)	15
Duration of Impella support, hours	265.5 ± 460.6	12
Duration of CS onset to Impella start, hours	15.6 ± 7.7	5
ICU stay, days	34.3 ± 46.7	15
Additional devices implanted/used during Impella support		
IABP	0 (0%)	15
ECMO	4 (26.7%)	15
VAD (CentriMag for RV support)	1 (6.7%)	15
**Hemodynamics during support**		
Heart rate, beats per minute	111 ± 35	7
Systolic blood pressure, mm Hg	111 ± 16	6
Diastolic blood pressure, mm Hg	88 ± 15	6
Mean arterial pressure, mm Hg	93 ± 12	6
Cardiac index, L/min/m^2^	2.73 ± 0.8	6
Cardiac output, L/min	5.31 ± 1.9	6
PCWP, mm Hg	14 ± 11	2
**Medications during Impella support**	9 (90%)	10
Beta‐blockers	1 (10%)	10
Calcium antagonists (Calcium channel blockers)	1 (10%)	10
ACE inhibitors	1 (10%)	10
Angiotensin receptor blockers	0 (0%)	10
Diuretics	5 (50.0%)	10
**Patient status**		
Survived and discharged alive	13 (86.7%)	15
Patient recovered from hemodynamic instability and successfully weaned off Impella	8 (53.3%)	15
Patient was bridged to other assist devices	6 (40.0%)	15
LVEF at discharge, %	28 ± 18	11
LVEF at additional follow‐up, %	38 ± 17	5

*Note*: Data presented as mean ± SD, or n (%).

Abbreviations: ACE, angiotensin‐converting enzyme; ECMO, extracorporeal membrane oxygenation; IABP, Intra‐aortic balloon pump; ICU, intensive care unit; LVEF, left ventricular ejection fraction; NYHA, New York heart association; PCWP, pulmonary capillary wedge pressure; VAD, ventricular assist device.

All women presented with acute decompensated heart failure with New York Heart Association (NYHA) class III or IV, CS complicating PPCM was present in 86.7% (13/15), and two women were resuscitated from cardiac arrest (16.7%) (Table 3). At baseline, the mean arterial pressure (MAP) was 75.0 ± 18.7 mm Hg and LVEF was 14.7 ± 6.1% (Table 1).

Prior to Impella support, 78.6% patients (11/14) were supported with inotropes or vasopressors and three patients with an intra‐aortic balloon pump (IABP). Among the 15 women receiving Impella support, Impella 2.5 was used in 33.3%, Impella CP in 53.3%, and Impella 5.0 in 13.3% (Table 3). The timing of onset of CS to Impella support was 15.6 ± 7.7 hours and duration of Impella support was 265.5 ± 460.6 hours. Four women received biventricular unloading with VA‐ECMO and Impella and two of them received VA‐ECMO prior to Impella without significant improvement in hemodynamics necessitating concomitant therapy with Impella.

### 3.2 Hemodynamics during MCS

Hemodynamics improved following LV unloading with Impella devices. Among women with hemodynamic measurements available both before and during Impella support, MAP increased from 71.8 ± 18.8 to 91.5 ± 11.9 mm Hg (*P* = .001, n = 11) and median pulmonary capillary wedge pressure decreased from 32.2 ± 8.6 to 17.8 ± 8.6 mm Hg (*P* = .007, n = 6) (Table 4).

**Table 4:** Comparison of hemodynamics before and on Impella support in women with peripartum cardiomyopathy

Measurements	N	Pre‐support	On support	*P*‐value
Heart rate, beats/minute	12	113 ± 27	102 ± 29	.35
Mean arterial pressure, mm Hg	11	72 ± 19	91 ± 12	.001
Cardiac index, L/min/m^2^	2	2.2 ± 0.2	3.7 ± 0.2	n/a
Cardiac output, L/min	2	4.4 ± 0.0	7.6 ± 1.3	n/a
PCWP, mm Hg	6	32 ± 9	18 ± 9	.01

*Note*: Data presented as mean ± SD.

Abbreviations: n/a, not applicable; PCWP, pulmonary catheter wedge pressure.

### 3.3 Clinical outcomes

Survival to discharge was 86.6% (13/15) (Table 3). Recovery of native heart functioning occurred in 53.3% (8/15), 40.0% (6/15) were bridged to another ventricular assist device, and two patients expired in the hospital (one patient who was admitted with acute liver failure developed irreversible disseminated intravascular coagulopathy and another patient died of septic shock after bridged to an LVAD).

Among the 11 survivors to discharge with data available, the average LVEF increased from the baseline value of 15 ± 7% to 28 ± 18% at discharge (*P* = .04). Follow‐up at 6 months was available in five patients who were treated medically in addition to Impella support. Ejection fraction in these patients increased from 11 ± 2% at baseline to 38 ± 17% (*P* = .01).

No myocardial infarction or stroke occurred until discharge. Two of 15 women (13.3%) had bleeding requiring transfusion and three additional women (20.0%) received transfusion due to anemia (Table 5). Need for new renal replacement therapy, infection, and hypotension during support was observed in three women each. Hemolysis occurred in three patients (20.0%) and limb ischemia in one patient (6.7%) who also received ECMO support. Increase in device purge pressure was noted in one patient with no consequence as the patient was successfully bridged to a durable LVAD. As described previously,^9^ adherent thrombotic material was found at the impeller housing in one case after device explanation, although without evidence of thromboembolism. One device malfunction was reported in a woman supported with an Impella 5.0 beyond the approved duration of support of 6 days (error message of “Impella sensor fail” displayed on the console on day 20 of support). No intervention was required and the Impella support was successfully continued for 43 days when the patient was transitioned to a surgical VAD.

**Table 5:** Adverse events at discharge

Adverse event		N
Death	2 (13.4%)	15
Myocardial infarction	0 (0%)	15
CVA/stroke	0 (0%)	15
Anemia requiring transfusion	3 (20%)	15
Bleeding requiring surgery	0 (0%)	15
Bleeding requiring transfusion	2 (13.4%)	15
Hematoma	0 (0%)	15
Limb Ischemia	1 (6.7%)	15
Vascular complication requiring surgery	1 (6.7%)	15
Vascular complication without surgery	1 (6.7%)	15
Hypotension during support	3 (20%)	15
Device malfunction	1 (6.7%)	15
New renal replacement therapy required	3 (20%)	15
Hemolysis	3 (20%)	15
Thrombocytopenia	1 (6.7%)	15
Infection	3 (20%)	15
Cardiopulmonary resuscitation	1 (6.7%)	15
Ventricular arrhythmia	2 (13.4%)	15
Respiratory dysfunction/failure	1 (6.7%)	15

*Note*: Data presented as n (%).

## 4 DISCUSSION

PPCM is a pregnancy‐associated, idiopathic form of cardiac dysfunction that can lead to severe morbidity and mortality. The incidence of this condition is on the rise,^12^ and it is a leading cause of non‐obstetrical maternal mortality in the United States.^13^ Although the majority of women demonstrate either partial or complete recovery after the diagnosis, the adverse outcome with severe and lasting morbidity and mortality remain unacceptably high. A subgroup of patients, mostly with a large myocardial insult (Ejection Fraction (EF) < 30%) at the time of diagnosis demonstrate further deterioration leading to severe heart failure, cardiogenic shock, and mortality.^14^, ^15^, ^16^

A recent algorithm for the management for severe acute PPCM, suggests optimization of oxygenation using noninvasive or invasive ventilation in patients with hypoxemia followed by the use of inotropic support.^5^ Analysis of the German PPCM registry suggested an unfavorable effect of dobutamine (a β1‐adrenergic receptor agonist) in women with PPCM.^17^ All women treated with the drug required either cardiac transplantation or LV assist devices, while 95% women not receiving dobutamine had LV recovery without the need for mechanical support, despite a similar degree of LV dysfunction. A potential explanation to these findings was suggested by Stapel et al who showed that β1‐adrenergic receptor stimulation with isoproterenol in STAT3‐deficient cardiomyocyte induced energy depletion, oxidative stress, and cell death.^17^ These cellular changes may underline the dobutamine‐induced irreversible HF deterioration in PPCM patients who frequently display reduced cardiac STAT3 expression.^18^ European recommendations favor the use of levosimendan based on the study by Labbene et al in 28 women with refractory CS (due to PPCM in eight women), which demonstrated significant hemodynamic and LV functional improvement at 48 hours following levosimendan administration.^19^ Levosimendan, however, is not available in the United States. Also, a randomized trial evaluating the addition of levosimendan to conventional heart failure therapy vs conventional therapy alone in 24 women with PPCM in Turkey showed no improvement in outcome in the levosimendan group.^20^ Moreover, in the Survival of Patients with Acute Heart Failure in Need of Intravenous Inotropic Support (SURVIVE) study including 1327 non‐PPCM patients, there was higher incidence of atrial fibrillation, hypokalemia, and headache without reduction of all‐cause mortality with levosimendan compared to dobutamine.^21^ The use of inotropic drugs in patients with CS is often limited due to the development of tachycardia, hypotension, and arrhythmias.^21^, ^22^ In addition, these drugs should be used with caution during pregnancy as fetal safety is not known due to limited information.^23^

The recommendations of the PPCM working group for the management of severe PPCM also include considerations for the use of bromocriptine. The use of bromocriptine in addition to standard heart failure therapy in the attempt to block the detrimental effect of cleaved prolactin has been shown to increase the rate of LV recovery and reduce mortality in two randomized, open‐label trials conducted in Africa.^24^, ^25^ A recent randomized trial from Germany evaluating 1‐week vs 8 weeks of bromocriptine therapy in 63 women with PPCM found similar improvement in LVEF.^26^ The study, however, was limited by the lack of a control group not receiving bromocriptine. Also, no information is available regarding the effect of bromocriptine in women with PPCM and CS. In the present report, bromocriptine was used in five of the 15 included patients. Because of the small number of patients and the concomitant use of inotropes and MCS, it was not possible to evaluate the effect of the bromocriptine alone. Given the hypotensive and prothrombotic effect of bromocriptine, its use in women with CS complicating PPCM may be limited.^5^, ^9^, ^27^ Four of the women in this study presented with severe heart failure prior to the delivery. The management of a woman presenting with severe PPCM prior to fetal maturation is challenging. The European guidelines recommend immediate delivery,^5^ which may be associated with a high risk of fetal mortality or life‐long complications. While the well‐being of the mother is a top priority, in this condition, maternal stabilization may allow for delaying of delivery and improving fetal prognosis.^28^

Percutaneous MCS devices are increasingly used in the treatment of CS. The Impella devices are transvalvular micro‐axial flow pumps that entrain blood from the LV and pump it into the aorta in series, thus unloading the LV. These devices are a valuable tool in the management of CS due to their ability to maintain vital organ perfusion, decrease LV wall stress and myocardial oxygen consumption, thereby enhancing the likelihood of ventricular recovery.^6^, ^29^ In fact, multiple studies have demonstrated that early initiation of Impella support within 90 minutes of CS onset was associated with higher survival.^30^, ^31^, ^32^ An additional advantage of early Impella use in CS is the decrease in the use of inotropic agents, thus, reducing the effects of these cardiotoxic drugs.^33^ In comparison to the growing evidence base of benefits of the Impella support in CS due to myocardial infarct, very few reports exist on the use of Impella in CS due to PPCM. In this study, the majority of women were diagnosed with PPCM post‐delivery with a mean LVEF of 14%. This group of women was previously shown to have a low likelihood of recovery.^34^ In addition, most of the women in the present study were in CS and received mechanical ventilation and inotropic support. Despite the critically ill nature of these women, >85% survived to discharge and 1 in two women had recovery of their native heart function, demonstrating the potential of MCS with Impella for hemodynamic stabilization and as a bridge to recovery or implantation of a durable cardiac assist device in women with CS due to PPCM.

Published case reports also suggest the potential stabilization of patients with CS due to PPCM with VA‐ECMO.^35^, ^36^ In spite of the success demonstrated with the use of VA ECMO, the benefit of this device needs to be carefully weighed against a high risk of complications including limb ischemia, stroke, acidosis, bleeding, thrombosis, infections, compartment syndrome resulting in muscle necrosis, lower limb ischemia that can lead to amputation, and Harlequin syndrome which may result in upper body and brain ischemia.^37^, ^38^ Surgical or catheter‐based insertion of a reperfusion catheter is often necessary to prevent limb ischemia. In addition, concomitant LV unloading is often required during VA‐ECMO support due to an increase in LV afterload leading to LV distension and increased LV filling pressure.^39^ In contrast to VA‐ECMO, a relatively low incidence of manageable adverse events was observed with the use of the Impella device in the 15 patients reported in this study.

### 4.1 Limitations

Limitations of this study include missing data in some patients, lack of a control group, and the use of multiple and non‐uniform therapeutic interventions, all inherent to any retrospective registry‐based analysis. Given the observational nature of this study, modest series of patients, and the concomitant use of inotropes, bromocriptine and multiple MCS, it was not possible to evaluate the effect of the Impella alone and hence the results should be considered hypothesis‐generating. Since PPCM is a rare disease and CS complicating PPCM occurs in very few women, the results of this study provide valuable insights into the use of MCS in this group of critically ill women.

## 5 CONCLUSIONS

To the best of our knowledge, this report is the largest series to date of hemodynamic support with Impella heart pump in women with CS complicating PPCM. The results of this study are encouraging and demonstrate that the use of Impella for LV unloading can be used for hemodynamic stabilization and as a bridge to recovery or successful implantation of durable MCS in patients with severe myocardial insult and CS due to PPCM.

## CONFLICT OF INTEREST

Uri Elkayam, Andreas Schäfer, Alaide Chieffo, Alexandra Lansky, Shelley Hall, Cindy Grines received speaker honorarium/consulting fees from Abiomed Inc. (Danvers, MA). Alexandra Lansky and Andreas Schäfer received institutional research funding from Abiomed Inc. (Danvers, MA). Zoltan Arany has no relevant disclosures.
